# Evaluation of multiple data sources for predicting increased need for HIV prevention among cisgender women: understanding missed opportunities for Pre-exposure Prophylaxis (PrEP)

**DOI:** 10.1186/s12879-023-08719-6

**Published:** 2023-11-09

**Authors:** Amy K. Johnson, Samantha Devlin, Sadia Haider, Cassandra Oehler, Juan Rivera, Isa Alvarez, Jessica Ridgway

**Affiliations:** 1 Reasearch Associate Professor Center for Gender, Sexuality, and HIV Prevention, The Potocsnak Family Division of Adolescent and Young Adult Medicine, Ann & Robert H. Lurie Children’s Hospital of Chicago, Northwestern University Feinberg School of Medicine, Chicago, IL USA; 2 Research Coordinator, University of Chicago, Chicago, IL USA; 3 Division of Family Planning, Rush University Medical Center (RUMC), Chicago, IL USA; 4 Clinical Assistant Professor Allegheny Health Network, Drexel University School of Medicine, Pennsylvania, USA; 5 Social and Behavioral Research Manager, Howard Brown Health, Chicago, USA; 6 Clinical Research Coordinator, Division of Family Planning, Rush University Medical Center (RUMC), Chicago, IL USA; 7 Biological Sciences Division, University of Chicago, Chicago, IL USA

**Keywords:** Pre-exposure prophylaxis, Women, HIV, Data sources HIV prevention

## Abstract

**Background:**

Ciswomen constitute a disproportionately low percentage of pre-exposure prophylaxis for HIV prevention (PrEP) users compared to men. Despite PrEP’s effectiveness, women are 5.25 times less likely to take PrEP than men. Identifying women who have increased reasons for HIV prevention and educating and offering PrEP to these women is crucial to reducing HIV transmission and overall health equity. However, the best method of identifying women at highest risk of acquiring HIV remains unknown. This study aimed to identify common HIV risk factors and data sources for identifying these common factors (e.g., electronic medical record data, open source neighborhood data), as well as potential intervention points and missed opportunities for PrEP linkage.

**Methods:**

We conducted an evaluation of multiple data sources: semi-structured qualitative interviews, electronic medical record (EMR) chart abstraction, and open source data abstraction. We accessed EMRs for enrolled participants and all participants signed a standard release of medical information (ROI) form for all institutions at which they had received medical care for the five-year period preceding their HIV diagnosis. Data were abstracted using a standardized procedure. Both structured and unstructured fields (i.e., narrative text of free notes) within the EMR were examined and included for analysis. Finally, open data sources (e.g., STI cases, HIV prevalence) were examined by community area of Chicago. Open data sources were used to examine several factors contributing to the overall Economic Hardship Index (EHI) score. We used these calculated scores to assess the economic hardship within participants’ neighborhoods.

**Results:**

A total of 18 cisgender women with HIV participated in our study. Participants were mostly Black/African American (55.6%) and young (median age of 34). Our analysis identified two main themes influencing HIV risk among participants: contextual factors and relationship factors. Further, potential pre-diagnosis intervention points and missed opportunities were identified during reproductive health/prenatal visits, behavioral/mental health visits, and routine STI testing. Our evaluation of multiple data sources included investigating the presence or absence of information in the EMR (STI history, HIV testing, substance use, etc.) as well as whether pertinent information could be gathered from open access sources.

**Conclusion:**

Ciswomen recently diagnosed with HIV identified many shared experiences, including syndemic conditions like mental illness and substance abuse, sex with men who have sex with men, and frequent moving in areas with high HIV incidence prior to their diagnosis. It is imperative that providers ask patients about social history, information about partners, and other key variables, in addition to the standardized questions. Findings can be used to better recognize ciswomen most vulnerable to HIV and offer PrEP to them, reducing HIV transmission.

**Supplementary Information:**

The online version contains supplementary material available at 10.1186/s12879-023-08719-6.

## Background

Cisgender women account for approximately 20% of annual new HIV diagnoses in the United States [1]. Incidence among women has plateaued and remained steady over the past decade [2]. Challenges across social-ecological levels increase HIV risk among subpopulations of women, including individual-level (low level of perceived HIV risk), interpersonal (unknown status of partner; risk of exposure during vaginal or anal sex; and intimate partner violence (e.g., women may be forced to have sex without a condom or medicines to prevent HIV)), institutional (lack of access to PrEP), and community (racism, discrimination, and HIV stigma) [1]. The complex interplay of these factors presents a considerable obstacle for screening tools used by providers to accurately identify women who have increased reasons for HIV prevention and, therefore, need pre-exposure prophylaxis (PrEP). PrEP is proven highly effective at preventing HIV, yet cisgender women (ciswomen) are known to be under-prescribed PrEP.

Ciswomen constitute a disproportionately low percentage of PrEP users compared to men [3]. In 2018, the PrEP-to-need ratio (number of PrEP prescriptions divided by number of new HIV diagnoses) for women (1.6) was less than a third of than for men (5.7), indicating a substantial inequity in PrEP use among ciswomen compared to their need [4]. Despite PrEP’s effectiveness as an HIV prevention method for high-risk women, women are 5.25 times less likely to take PrEP than men [5]. Challenges exist at each step of the PrEP care continuum, starting with difficulty identifying ciswomen at highest risk of HIV who are the most likely to benefit from PrEP [6], followed by low self-perception of HIV risk [7, 8], low PrEP knowledge [9, 10], and structural and individual barriers to PrEP initiation and persistence [11, 12]. Although there has been growing knowledge of interventions to increase uptake of PrEP among men who have sex with men (MSM) and transgender women [13–15], much less is known about successful strategies to increase PrEP uptake among ciswomen. Moreover, studies investigating barriers and facilitators to PrEP among ciswomen typically include women who are HIV-negative rather than those who have already been diagnosed with HIV [16–18], creating a gap in knowledge of valuable perspectives that can help determine how to identify women who are most vulnerable to HIV and in need of PrEP.

To reduce HIV transmission and contribute to health equity, subpopulations that face the highest risk of HIV acquisition must use PrEP according to their need. Identifying women who have increased reasons for HIV prevention and educating and offering PrEP to these women is crucial. However, the best method of identifying women at highest risk of acquiring HIV remains unknown. This study aimed to ascertain common HIV risk factors and data sources for identifying these common factors (e.g., electronic medical record data, open source neighborhood data), as well as potential pre-diagnosis intervention points, and missed opportunities for PrEP linkage among ciswomen recently diagnosed with HIV, as these patients can retrospectively define a “high risk” group and provide valuable insight to further refining screening tools for identifying ciswomen PrEP candidates in the future.

## Methods

We conducted an evaluation of multiple data sources: semi-structured qualitative interviews, electronic medical record (EMR) chart abstraction, and open source data abstraction. Women who received HIV related medical care at the University of Chicago Medicine (UCM) and Howard Brown Health (HBH) were recruited to participate in the study. Women were eligible for the study if they were (1) aged 18 years or older; (2) female sex at birth and current gender identity of female; (3) English speaking; (4) diagnosed with HIV within the past 5 years from the date the interview occurred; and (5) able and willing to provide informed consent.

A semi-structured interview guide was developed and used to capture information related to common HIV risk factors and potential pre-diagnosis intervention points [see Additional file 1]. Before the COVID-19 pandemic (December 2019-March 2020), 10 interviews were conducted in-person with patients from UCM by trained research staff in a private, secure location. All research staff were cisgender women. During the COVID-19 pandemic (April 2020-April 2021), 8 interviews were conducted remotely via telephone. Of note, the COVID-19 pandemic severely impacted recruitment efforts. All participants provided informed consent, with remote interviewees using REDCap software to sign an electronic consent form. All interviews were audio recorded and then transcribed. This study was approved by the University of Chicago Institutional Review Board (19-1345) and Lurie Children’s IRB (21-4506). All research was performed in accordance with the Declaration of Helsinki.

Transcripts were analyzed using Dedoose, an online qualitative research software [19]. A preliminary codebook was developed from the interview guide with clear definitions for each code; all coders (N = 3) reviewed and revised the preliminary codes. Next, the codebook was applied by the primary coder to two transcripts, and secondary coders (N = 2) coded a subset of excerpts selected at random and achieved reliability with Cohen’s Kappa > 0.80. Most divergences occurred due to omission and upon review were quickly rectified to 100% agreement. Codes were then applied to all 18 transcripts by a primary coder and were reviewed by a secondary coder for consensus of code application. Each transcript was coded iteratively and examined for emergent themes. Interviews were analyzed using a deductive thematic content analysis approach for common factors related to potential pre-diagnosis intervention points and missed opportunities for PrEP [20]. Finally, themes were elicited based on clustering of code application, and representative quotes were selected to highlight salient themes. In addition to conducting interviews, we accessed EMRs for enrolled participants and asked all participants to sign a standard release of medical information (ROI) form for all institutions at which they had received medical care for the five-year period preceding their HIV diagnosis. We requested data from the EMR regarding their social history and history of sexually transmitted infection (STI) testing/treatment, HIV testing, and emergency room (ER) visits. If participants declined to release medical records, they were still offered to complete the interview and have their qualitative data contribute to the study. Data were abstracted using a standardized procedure. Both structured and unstructured fields (i.e., narrative text of free notes) within the EMR were examined and included for analysis.

Finally, open data sources (e.g., STI cases, HIV prevalence) were examined by community area of Chicago. Neighborhoods were either self-reported during qualitative interviews or were found using zip code listed in the participants’ EMR. HIV and other STI rates were examined by community areas of Chicago for 2019 using the Chicago Department of Public Health (CDPH) 2020 HIV/STI Surveillance Report [21]. These rates were analyzed and organized into community areas that ranked in the top 10% and top 25%, respectively, for each metric.

Open data sources were used to examine several factors contributing to the overall Economic Hardship Index (EHI) score. This score, which utilizes multiple indicators to provide a comprehensive view of economic hardship was calculated by the Great Cities Institute at the University of Illinois at Chicago for each community area in Chicago using data from 2013 to 2017 [22]. We used these scores to assess the economic hardship within participants’ neighborhoods.

## Results

Between December 2019 and April 2021, 18 ciswomen recently diagnosed with HIV in Chicago, IL participated in the study. As seen in Table 1, participants were mostly Black/African American (55.6%) and young (median age of 34). Neighborhoods were reported and confirmed by zip code within EMRs for 15/18 participants. Of those women, 14 resided in Cook County, Illinois with 11 participants living in the city of Chicago; 1 participant lived in Indiana.

**Table 1:** Participant Demographics

Variable	n (%)
**Age (Years)**	
Median Range IQR	34 23–72 25
**Race**	
Black/African American White Native American/Other Pacific Islander Unknown/Undetermined	10 (55.6%) 3 (16.7%) 1 (5.5%) 4 (22.2%)
Place of Residence*	
Chicago Other	11 (73.3%) 4 (26.7%)
**Neighborhood in Chicago** ^**Ŧ**^	
Chatham Woodlawn Englewood South Shore River North Uptown Roseland Rogers Park	1 (9.1%) 3 (27.2%) 2 (18.2%) 1 (9.1%) 1 (9.1%) 1 (9.1%) 1 (9.1%) 1 (9.1%)

*15/18 participants

**Ŧ**11/18 participants

Our study identified two main themes related to HIV risk among participants: contextual factors and relationship factors. Further, potential pre-diagnosis intervention points and missed opportunities for PrEP were identified during reproductive health/prenatal visits, behavioral/mental health visits, and routine STI testing. Consistent themes among patients are highlighted below. Our evaluation of multiple data sources included investigating the presence or absence of information in the EMR as well as whether or not pertinent information could be gathered from open access sources (Table 2).

**Table 2:** Triangulation of data sources representing common risk factors and potential pre-diagnosis intervention points and missed opportunities for PrEP

Common risk factors and missed opportunities for PrEP prior to HIV diagnosis	Self-reported data during qualitative interview (N=18) n (%)	Supporting documentation within the electronic medical record (EMR) n (%)
Had a male partner who had sex with other men (MSM) Had a male partner who had been previously incarcerated Had a mental health disorder Saw a mental health professional Had at least one previous sexually transmitted infection (STI) diagnosis	6 (33.3) 8 (44.4) 10 (55.6) 9 (90) 11 (61.1)	0 (0) 0 (0) 6 (10) 2 (22.2) 3 (27.3)
**Data reported during qualitative interviews (N=18)**	**n (%)**	
Moved at least once during the year prior to HIV diagnosis Had not heard of PrEP, were unaware PrEP was available for women, and/or were never offered PrEP by a healthcare provider	9 (50) 12 (66.7)	
**Data documented within the EMR (N=15)**	**n (%)**	
Current or previous employment status (i.e., descriptions of employment or unemployment) Current or previous housing instability/homelessness Current or previous drug use/abuse Current or previous alcohol use/abuse Relationship status Single Partnered Sexual history/activity (e.g., number of sexual partners, protected vs. unprotected sex) Prior HIV test results Date of last negative HIV test	13 (86.7) 13 (86.7) 8 (53.5) 11 (73.3) 15 (100) 14 (93.3) 1 (6.7) 11 (73.3) 7 (46.7) 6 (85.7)	
**Data elicited from open sources for Chicago (N=11)**	**n (%)**	
Lived in community area that ranked in the top 10% for HIV incidence and prevalence Lived in community area that ranked in the top 25% for HIV incidence and prevalence Lived in community area that ranked in the upper quartile for HIV incidence and prevalence Lived in community area that ranked in the top 25% for both chlamydia and gonorrhea incidence rates Lived in community area that ranked in the top 10% for primary and secondary syphilis incidence rate Lived in community area that ranked in the top 25% for unemployment rate for the population aged 16 and older Lived in community area that ranked in the top 25% of households with income below the poverty level Lived in community area that ranked in the top 10% of the calculated EHI score	3 (27.3) 6 (54.5) 9 (81.8) 8 (72.7) 3 (27.3) 5 (45.5) 6 (54.5) 2 (18.2)	

PrEP: Pre-exposure Prophylaxis. EMR: Electronic Medical Record. EHI: Economic Hardship Index

### Common HIV risk factors- contextual

Contextual factors that increased participants’ vulnerability to HIV included experiencing homelessness, engaging in survival sex, unemployment, and drug/alcohol use/misuse. In qualitative interviews, some women reported engaging in survival sex or sex work to support themselves, as this participant describes the intersection of her drug use and engagement in sex work:

“During the course of my alcohol and drug use, I was a sex worker. I worked the streets as a prostitute and that’s another reason why I, when I got diagnosed, I couldn’t really pinpoint how I contracted it [HIV].” (ID 3).

Some participants experienced housing instability and/or homelessness during the year of their HIV diagnosis:

“After I left high school, that’s when everything went downhill for a long time… of back and forth and just… I was homeless…even though I had places to go, I didn’t have my own.” (ID 7).

Further, nine (50.0%) women reported moving at least once during the year prior to their HIV diagnosis indicating an increased level of housing mobility. Participants also described periods of un- or underemployment, difficulty with making “ends meet”, and the impact of drug/alcohol use on their sexual behavior and relationships.

Evaluating EMR data, 11/15 (73.3%) participants had documentation of either current or past alcohol use/abuse. Just over half of participants (53.3%) had descriptions of either current or past drug use/abuse (including marijuana) within their EMR. Engagement in sex work was documented for one patient in free-text notes. Employment status, including descriptions of employment or unemployment both prior to and after HIV diagnosis, and information regarding housing instability or homelessness was found within the EMR for 13/15 (86.7%) women.

Reflective of participant’s narratives, using open source data, 18.2% of participants lived in community areas of Chicago that ranked in the top 10% of the economic hardship index. Six (54.5%) women lived in community areas that ranked in the top 25% of households with income below the poverty level. Five (45.5%) participants lived in community areas that ranked in the top 25% for unemployment rate for the population aged 16 and older within Chicago.

### Common HIV risk factors- relationship

Women described their sexual and romantic relationships during the time period in which they were exposed to HIV. These relationships often had characteristics that increased their risk for HIV acquisition. One of these characteristics was control and violence within intimate partner relationships:

“I didn’t know how to get away, and the more-the more I would- the longer I was with him, the harder it became to find my way out. I became dependent because I pushed everybody off- out of my life.” (ID 18).

At times, participants described physical abuse due to their partner’s drug and alcohol use:

“He had become violent. He had to stop- he was using. He was smoking cocaine, smoking crack, and that was dangerous for me…” (ID 3).

“When I did feel unsafe, I called the police. Because we had fights. We had fights. Because he, of course, was an alcoholic too [in addition to his family member]. And, he…he gets angry when he drinks and when he can’t have things his way.” (ID 6).

Participants also described relationships in which their male partners had multiple other sex partners and/or were having sex with other men:

“I was feeling good. And looking for next steps, what to do…I just met a guy and kind of really, really fell for him. Looking back, I see all the people he hung out with were LGBT. And yeah, I knew he had, uh, a husband.” (ID 11).

“Yeah, he’s bisexual. That I know. I found that out too.” (ID 18).

“I actually caught him with, um, like, five other girls.” (ID 15).

Finally, participants shared that their male partners were incarcerated or had histories of incarceration:

“He’s currently incarcerated right now, and he was incarcerated before I met him.” (ID 14).

In the EMR data, of the 15 participants who had available data, 100% had descriptions of their relationship status. Fourteen (93.3%) were listed as single and 1 (6.7%) was listed as partnered. The majority of participants (11/15; 73.3%) also had documentation regarding their sexual history/activity (e.g., number of sexual partners, protected vs. unprotected sex). Of the 18 participants, 6 (33.3%) women reported having a male partner who had sex with other men (MSM), and 8 (44.4%) participants reported having a sexual partner who had been incarcerated previously. None of this information about partners’ MSM sexual activity or prior incarceration was documented within the EMR.

Overall, 11/18 (61.1%) participants had at least one previous STI diagnosis. Of these 11 women, 2 (18.2%) were documented in the EMR alone and not reported during the interview, 1 (9.1%) was both documented in the EMR and reported during the interview, and 8 (72.7%) were only reported during the interview and missing from the EMR records we reviewed.

### Potential pre-diagnosis intervention points and missed opportunities for PrEP linkage

We identified several pre-diagnosis intervention points that could increase access to PrEP and support uptake among ciswomen. Participants were generally engaged in routine healthcare and sexual health screenings. Many women felt that healthcare providers should talk to patients about PrEP, either in reproductive health visits, as this participant describes:

“I think when they [women] go to get their physicals, like when they go to see the gynecologist for their Pap smear and stuff like that, they should be offering [PrEP] and someone should stay with them and tell them like, ‘This is to prevent, you know, this, from getting HIV.’” (ID 6).

or during routine healthcare visits, as these participants describe:

“I feel like, uh, more healthcare providers should bring it [PrEP] up, or you know, recommend it more and maybe…more women will be aware to it because I’m one of those women that was not aware to it at all, until it was too late.” (ID 12).

“I don’t see why doctors wouldn’t just see it as part of their job to do it [offer PrEP], as they see as part of their job to offer all of these other medications and vaccines; like, it’s just another one added to the list of other things to give to their patients. Like, I don’t see why they can’t just add it.” (ID 15).

Close to half of the participants (7/15; 46.7%) had prior HIV test results within their EMR, with 6/7 (85.7%) listing the date of their last negative test. Overall, 10/18 (55.6%) participants had at least one mental health disorder prior to their HIV diagnosis; 6/10 (60%) were both documented in the EMR and reported during the interview, and 4/10 (40.0%) were only reported during the interview and missing from the EMR. Overall, 9/18 (50.0%) women saw a mental health professional prior to their HIV diagnosis. Of these 9 women, 2 (22.2%) were both documented in the EMR and reported during the interview, and 7 (77.8%) were only reported during the interview and missing from the EMRs that were reviewed.

For the 11 women living in Chicago, 6 (54.5%) participants resided in community areas that ranked in the top 25% for both HIV incidence and HIV prevalence rates within Chicago in 2019. Eight (72.7%) participants lived in neighborhoods that ranked in the top 25% for both chlamydia and gonorrhea incidence rates. Three (27.3%) women resided in community areas that ranked in the top 10% for primary and secondary (P&S) syphilis incidence rate for Chicago in 2019.

In discussing PrEP awareness during the qualitative interviews, 12 women (66.7%) reported they had not heard of PrEP prior to their diagnosis, expressed that they were unaware that PrEP was available for women to take, or were never offered PrEP by a healthcare provider prior to their diagnosis. One participant articulated her perceptions that PrEP is not often discussed among straight people and talking about PrEP is more acceptable in gay communities:

“I associate PrEP more, and I’m gonna be honest, with gay men. It’s not something I think that’s brought up in the straight community between, you know, a woman and a man that’s in, you know, in that type of relationship…when I hear people talking about PrEP or when I see commercials, or when I see things, I feel like it’s more associated towards, um, the gay community, to be honest.” (ID 18).

## Discussion

Our results highlight missed opportunities for prescribing PrEP to women as well as ways we can better capture data in the EMR to guide automated alerts of women with indications for PrEP. Information provided during qualitative interviews with women such as partners’ incarceration history, partners’ sexual activity with other men, and previous STI diagnoses, were absent from the majority of participants’ EMRs that were available. There are clearly gaps in documenting relevant relationship and contextual information that impacts HIV risk within the EMR. Additionally, use of existing data within the EMR, such as address and community area, could better inform provider action. Prior studies have documented how many people living with HIV (PLWH), particularly ciswomen, experience missed opportunities to receive PrEP [6, 23, 24]. The women in our study were engaged in the healthcare system, with the majority reporting and/or having evidence within the EMR of routine STI screening, engaging in mental health services, receiving prenatal care, and receiving reproductive health screenings (e.g., Pap smears); however, despite being engaged in the healthcare system, the majority of women were not offered PrEP as an HIV prevention tool or were unaware that PrEP existed and was available for them to take, as reported during the qualitative interviews.

In a recent study assessing PrEP eligibility (based on prior CDC guidelines) in emergency room (ER) patients, results indicated MSM and heterosexual men were more likely to meet PrEP eligibility based on EMR data compared to women due to limited patient-reported information on sexual behavior of partners, partner’s HIV status, and partner’s condom use [25]. This study reinforces our findings that additional information may need to be documented to accurately assess HIV risk among women. Furthermore, many of the characteristics known to increase vulnerability to HIV are not routinely tracked and were not documented in the EMR but were only elucidated through qualitative interviews. This represents an opportunity to refine EMR tools to support automated identification of PrEP-indicated patients and ensure comprehensive sexual histories include contextual factors as well as partner characteristics.

There is substantial evidence that social and contextual factors impact health outcomes. Indeed, the Institute of Medicine recommends that Social Determinants of Health (SDoH) indicators (e.g., social connection, intimate partner violence, stress, mental health) be documented in the EMR [26, 27]. However, studies have found that SDoH data are often poorly documented in the EMR, subject to biases and missing data [28–30]. In our study, we found that SDoH data, which could potentially inform risk for HIV, were often missing from participants’ EMRs. Barriers to collecting SDoH data during clinical care include lack of provider time, challenges integrating SDoH screening into clinic workflow, and staff discomfort discussing sensitive information with patients [31]. Standardization of EMR SDoH documentation and utilization of patient self-reported data are strategies that have been found to improve SDoH documentation [27]. Similar strategies could be utilized to collect data specific to HIV vulnerability among women (e.g., behaviors of sexual partners).

In addition to individual-level SDoH data, community-level data can also provide valuable insight into social context that can impact health. Patients’ addresses can be geocoded and matched to publicly available data regarding SDoH in their community area (e.g., median income, financial hardship, etc.) [32]. We found that many of the participants in our study lived in neighborhoods with high economic hardship as well as high incidence of HIV and other STIs. Matching patient address to community area and associated contextual socio-structural data could occur via an automated process, thereby providing contextual information regarding patients’ SDoH and HIV risk, with minimal extra effort required from clinic staff.

The strengths of this study include the recruitment of women who were diagnosed with HIV within the past 5 years and the evaluation of multiple sources of data. However, study results need to be interpreted considering some limitations. Participants were recruited from two clinics with substantial HIV care programs in an urban setting in the Midwest and were limited to those who spoke English; thus, our findings may not reflect the experience of recently diagnosed women who receive HIV care at smaller or rural healthcare clinics or those who do not speak English. In addition, the COVID-19 pandemic emerged in the middle of our study period, thereby minimizing recruitment efforts and potentially impacting patients’ willingness or ability to participate. Although the total sample size is relatively small (N = 18), we achieved theme saturation and generated several consistent themes across the dataset. Various sources of inevitable bias associated with qualitative research may have impacted study findings, including the impact of interviewer identity. Women who have better engagement in the healthcare system may have been more likely to participate than others, and interviewer presence may have impacted participant responses or the selection of salient themes. We did not ask participants to reflect on their experiences with the interviewer. However, establishing inter-rater reliability diminished the effects of bias during qualitative data analysis and led to consensus on code application and elicitation of significant themes. Additionally, the COVID-19 pandemic also limited our ability to access all medical records. Some women experienced homelessness and others did not visit a health care site within the past few years. It is possible that the missing information is located within EMRs that were not reviewed or would be more easily found in the EMRs of women who are well engaged with the healthcare system. Future research should continue to investigate the most accurate way to identify ciswomen who are at highest risk for HIV and thus most likely to benefit from PrEP. Findings from this study highlight the urgent need for research with larger sample sizes. Future studies should evaluate modifying the existing PrEP identification screening tools within healthcare systems to better identify at-risk women and provide them with HIV prevention resources including education and PrEP. Overcoming the barriers to allow more women to access PrEP would significantly help to reduce HIV transmission among ciswomen.

## Conclusion

Ciswomen recently diagnosed with HIV identified many shared HIV risk factors, including syndemic conditions like mental illness and substance abuse, sex with MSM, and frequent moving in areas with high HIV incidence prior to their diagnosis. As automated algorithms rely on data found within EMRs, it is imperative that providers ask patients about their social history, information about their partners, and SDoH variables, in addition to standardized questions within structured EMR fields. These findings can be used to modify electronic risk identification systems to better recognize ciswomen who are most vulnerable to HIV and offer PrEP to them, thereby reducing HIV transmission among ciswomen.

### Electronic supplementary material

Below is the link to the electronic supplementary material.

Supplementary Material 1

## Data Availability

The datasets used and/or analyzed during the current study are available from the corresponding author on reasonable request.

## Abbreviations

HIV: Human Immunodeficiency Virus
PrEP: Pre-Exposure Prophylaxis
EMR: Electronic Medical Record
HBH: Howard Brown Health
UCM: University of Chicago Medicine, COVID-19:Coronavirus Disease 2019
STI: Sexually Transmitted Infection
ER: Emergency Room
CDPH: Chicago Department of Public Health
EHI: Economic Hardship Index
MSM: Men Who Have Sex With Men
PLWH: People Living With HIV
SDoH: Social Determinants of Health
