# Supplementary material for: Evaluation of multiple data sources for predicting increased need for HIV prevention among cisgender women: understanding missed opportunities for Pre-exposure Prophylaxis (PrEP)
Source: BMC Infect Dis. 2023 Nov 9;23:781. doi: 10.1186/s12879-023-08719-6 (PMC10636899; doi:10.1186/s12879-023-08719-6)
Supplement: Supplementary file 1 — Supplementary Material 1 [file 12879_2023_8719_MOESM1_ESM.pdf]

File name: Additional file 1
File format: .doc
Title of data: Interview Guide
Description of data: A semi-structured interview guide with questions designed to capture information related to common HIV risk factors and potential pre-diagnosis intervention points

PROTOCOL TITLE: Our stories, our lives, our health: Refining an automated identification of HIV-negative, PrEP-eligible women in the emergency department

Interview Objectives

- Understand the context and life experiences of participants, including exposure to HIV prevention messaging, social stressors, and other characteristics that may identify potential intervention points for PrEP linkage
- Analyze data to identify potential intervention points and missed opportunities for PrEP linkage

PART 1. INTRODUCTION

- Quick review
    - We are going to be asking a lot of questions about your life experiences, particularly leading up to your HIV diagnosis. If there are any questions you would rather not answer, please let me know and we can move on.
    - Audio recording is for research purposes and for internal use only. When we are done, someone will type out every word that was said. After that, we will destroy the recordings so that everyone's participation is anonymous.
    - We will be asking lots of questions. There are no right or wrong answers, we are interested in your opinions and your experiences. After respond, we may ask you "why" – not because we disagree, but to make sure we understand your thoughts and capture all your ideas.

Did you have any questions before we begin?

I'll be taking notes during the interview. However, with your permission, I'd also like to audio-record our conversation as I may not be able to write down everything quickly enough. The audio recordings will be destroyed at the end of the study.

Do I have your permission to record our conversations? YES NO

PART 2. CALENDAR

The first thing we are going to do is talk through this past year, as I'm going to be asking a lot of questions about this past year. So today is DATE of the YEAR. I'm going to mark the month right here. Now, let's add some events to help you think about the past year- when is your birthday? How about Halloween or Valentine's day? Did you have any family or friends visit at any time this past year? Let's also include the date you were diagnosed with HIV. Good, let's mark all these events on our timeline. We will be talking about the past year that is from today, all the way back to DATE.
Past year

January : ________________
February: ________________
March : ________________
April: ________________
May : ________________
June : ________________
July: ________________
August: ________________
September: ________________
October : ________________
November: ________________
December: ________________

PART 3. HEALTH CARE EXPERIENCES

Ok, the first thing I want us to talk about is health care. I'm going to ask you some questions about accessing health care before you were diagnosed with HIV (Show on timeline). First, we'll talk about health care in general then we'll get a little more specific and talk about mental health care.

1. Prior to being diagnosed with HIV, did you get care with a health care provider? What kind of provider? *Can clarify- if not PCP, a specific clinic or urgent care?*

   Where do you go (or who did you see) when you had a health problem?
   a. *Probe: regular source of care, how you choose for what*
   b. *Reproductive health needs-family planning/ sexual health; ER (list times & places of use- specific names)*
      i. *How many times have you been pregnant? How many births?*
      ii. *What do you use for family planning (birth control)?*

2. Did you discuss sexual health or HIV prevention at any of your general health care appointments?
      i. If yes, describe what you discussed; how often did these discussions occur?
      ii. If no, did you want to talk about HIV prevention or sexual health? What were the barriers? What could have made it easier?

3. Have you ever been tested for STDs, like chlamydia or gonorrhea? Tell me about the most recent time you received an STD test.
   Probe: where, what was the outcome, HIV prevention messaging, why did you get tested (symptoms or…)? any behavior change, discuss with partner?

4. Prior to being diagnosed with HIV, how often did you get tested for HIV?
   a. Where did you get tested?
   b. When was the last time you were tested, prior to your diagnosis?
   c. What prompted you to get tested?

5. Prior to being diagnosed with HIV, did you see a provider for concerns about your mental health? When I say mental health, I mean how you are feeling emotionally. …
   a. If yes, can you tell me more about it?

i. *General experience, trust, relationship, outcome; can probe for HIV prevention messaging*

b. If no, have you considered seeing a provider for mental health reasons? What prevented or made it hard for you to seek mental health care?

PART 4. SOCIAL CHARACTERISTICS

6. In the past year, how many neighborhoods have you lived in? Which neighborhoods?

7. In the past 6 months, (that is since CALENDAR reference) was there ever a time when you did not have a place to stay or to sleep?
   a. Can you tell me about the different places you have stayed or slept in the past year?

8. Are you currently employed or looking for employment? Can you tell me a little about your job or about the job you are looking for?

9. In the year prior to your HIV diagnosis, did you feel safe in your home (where you were living)? Can you tell me more about why you felt this way.

10. Sometimes people use alcohol or drugs, can you tell me about your use of alcohol and/or drugs in the year prior to your HIV diagnosis (review CALENDAR)?
    a. Did you inject drugs?
    b. Was your drinking or drug use ever considered a problem by you or others?
    c. Did you seek treatment for drug or alcohol use?

PART 5. ROMANTIC RELATIONSHIP, HIV PREVENTION EXPOSURE, & PrEP

11. Can you share with me how you were infected with HIV- was it through sex or injection drugs or some other way?

12. Can you tell me a little about the sexual partners you were with during the time when you think you were infected with HIV? What was your relationship(s) like? Did your partner disclose their HIV status?

13. Prior to being diagnosed, what were your HIV prevention strategies?
    Condoms, partner status, etc.

14. How concerned were you about HIV? Can you tell me more about this? What influenced your feelings about your HIV risk?

15. Prior to learning you are living with HIV, had you ever heard of PrEP – a daily medication that is used to prevent HIV-negative people from getting HIV?

16. Before you were diagnosed, were you ever offered PrEP by a medical provider?

If yes, who offered you PrEP the first time?

Probe: outreach worker, community-based organization staff, friend, sex partner

Did you ever take PrEP?
What do you think about PrEP as an option to prevent HIV infection?

- *Probe: effectiveness, convenience, versus condom use*

Do you know anyone on PrEP? Who are they?
How many of your close friends, family members or sex partners are on PrEP?

What do your close friends say about PrEP?
Probe: stigma, acceptance, lack of knowledge; what about other cis women?
How is PrEP perceived by your friends? Family members?
How is PrEP perceived in your community?

17. We know that more women should be on PrEP. What do you think are the biggest barriers to getting on PrEP for women? How do you think we can overcome these barriers?

PART 6. COVID-19

18. In this next part of the interview we are going to ask a few questions about COVID-19, also called the coronavirus. On March 12, the World Health Organization declared COVID-19 a pandemic.

19. We know that everyone's lives are different now. I'm curious to understand how the COVID-19 pandemic has impacted your life?
Probes: financial impact; childcare responsibilities; mental health; physical health; relationship strains;

20. How has the COVID-19 pandemic impacted your ability to remain in HIV care?
Probes: difficulty accessing medication; fear about attending medical appointments; depression; anxiety; financial strain

21. How has the COVID-19 pandemic impacted your sexual behavior and romantic relationship(s)?
Probes: having sex less often or more often; being away from partners; not having access to birth control/condoms; unable to meet anonymous partners/increased meeting of anonymous partners

22. How concerned are you that you will contract COVID-19?

23. Can you talk me through what you would do if you had symptoms of COVID-19?
Probes: any fears/concerns about unjust treatment when accessing/attempting to access the healthcare system?

Conclusion and Thank You
Thank you for your time and for sharing your thoughts, experiences and feelings with us. You will receive your incentive after you fill out this receipt.
